# A new method of adjusting mesh tension using cystoscopy during laparoscopic sacrocolpopexy

**DOI:** 10.1007/s00192-021-04791-1

**Published:** 2021-04-19

**Authors:** Yukiko Nomura, Yoshiyuki Okada, Aya Hiramatsu, Eiji Matsubara, Kumiko Kato, Yasukuni Yoshimura

**Affiliations:** 1 Department of Female Pelvic Health Center, Showa University Northern Yokohama Hospital, 35-1, Chigasakichuo, Tsuzuki Ward, Yokohama City, Kanagawa Prefecture 224-8503 Japan; 2 Department of Obstetrics and Gynecology, Showa University Northern Yokohama Hospital, Yokohama, Kanagawa Japan; 3 Department of Urology, Showa University Northern Yokohama Hospital, Yokohama, Kanagawa Japan; 4 Department of Female Urology, Japanese Red Cross Nagoya First Hospital, Nagoya, Aichi Japan

**Keywords:** Cystoscopy, Laparoscopic sacrocolpopexy, Pelvic organ prolapse, Stress urinary incontinence

## Abstract

We aimed to examine the usefulness of cystoscopic findings to guide mesh tension adjustment during laparoscopic sacrocolpopexy (LSC) to promote better repair of pelvic organ prolapse (POP) and prevent de novo stress urinary incontinence (SUI). In this technique, the bladder wall was observed using a cystoscope when various traction pressures were applied by pulling the mesh arm with forceps before fixation to the promontory during LSC. Adjustment was performed on 20 patients, and postoperative outcomes of POP repair and development of de novo SUI were evaluated. When excessive traction was applied on the mesh arm, a bladder neck opening and a cord-like elevation in the center of the trigone and posterior wall were observed in all cases. The tension was gradually loosened, and precisely when the above-mentioned cystoscopic finding (“Central Road”) disappeared, an anatomically appropriate elevation of the vaginal apex was achieved; the mesh arm was fixed to the promontory. At 6 months after LSC, anterior wall recurrences were diagnosed in four patients (beyond the hymen in one) with few symptoms, while no occurrence of de novo SUI. Cystoscopic findings during mesh tension adjustment in LSC could be useful in achieving improved POP repair.

**Supplementary Information:**

The online version contains supplementary material available at 10.1007/s00192-021-04791-1.

## Introduction

Laparoscopic sacrocolpopexy (LSC) is considered an excellent option for apical vaginal prolapse owing to its superior correction rates in treating pelvic organ prolapse (POP) [1]. However, de novo stress urinary incontinence (SUI) is a potential complication that occurs with a high incidence ranging from 7.5% to 26% in women who have undergone LSC [2–6].

A greater reduction in point Aa increased the risk of de novo SUI [2]. Since no objective indicators have been identified and few reports have described mesh tension adjustment during LSC [7], excessive traction pressure may be inadvertently applied on the LSC mesh to achieve sufficient prolapse repair.

Kato et al. recently reported an unusual cystoscopic finding in a woman with severe mixed urinary incontinence following LSC [8]. A cord-like appearance caused by excessive tension on the LSC mesh was revealed in the center of the bladder trigone and posterior wall, and it was named the “central road” (CR). Applying undue pressure on the LSC mesh toward the sacrum may cause excessive straightening (de-kinking) of the bladder neck and proximal urethra, subsequently increasing SUI. Thus, this study aimed to examine the usefulness of cystoscopic findings to guide mesh tension adjustment during LSC to promote POP repair and prevent de novo SUI. We hypothesized that adjusting the mesh tension to avoid the CR finding and bladder neck opening on cystoscopy could prevent de novo SUI following LSC.

## Method

Twenty women with symptomatic stage 3–4 POP according to the Pelvic Organ Prolapse Quantification (POP-Q) system underwent LSC with mesh tension adjustment under cystoscopic guidance at our center from July 2019–April 2020. The POP-Q was used for objective prolapse assessment before and 3 and 6 months after surgery. Prolapse recurrence was defined as retreatment (pessary use or surgery) or POP-Q stage ≥ 2 prolapse. Prolapse Quality of Life Questionnaire (P-QOL) [9] was used to evaluate the mental and physical state of patients, and the International Consultation on Incontinence Questionnaire for Urinary Incontinence Short Form (ICIQ-UI SF) [10] was used to subjectively assess SUI; these evaluations were performed before surgery and 3 and 6 months after surgery. Preoperative occult SUI evaluation was performed via an interview form and a stress test with prolapse reduction using a gauze or pessary in the lithotomy position. The occurrence of postoperative de novo SUI was assessed in women without formerly (before surgery) diagnosed overt or occult SUI.

The study was conducted as per the principles of the Declaration of Helsinki and approved by the Institutional Review Board (approval number: 19H046). Informed consents were obtained from all patients.

### Surgical technique

All surgical procedures were performed by two senior surgeons according to the procedure by Wattiez et al. [7]. A polytetrafluoroethylene (PTFE) type 1 macropore polyfilament mesh (ORIHIME®, CROWNJUN, Kono Seisakusho Co., Ltd., Chiba, Japan) was used. The patient was placed in a Trendelenburg (15º) and lithotomy position under general anesthesia. A pneumoperitoneum was created with the intra-abdominal pressure set at 10 mmHg. Subtotal hysterectomy and salpingectomy were performed. Either oophorectomy or ovarian preservation was performed according to the patient’s wishes. The anterior vaginal wall was dissected from the bladder till the bladder neck level. The anterior mesh was fixed to the anterior vaginal wall in 3–6 places with polyester #3/0 non-absorbable sutures and to the cervix with polyester #2/0 sutures. The posterior mesh was fixed bilaterally to the levator ani with polyester #2/0 sutures and to the perineal body with polyglactic #2/0 absorbable sutures. The anterior and posterior meshes, including the bilateral uterosacral ligaments, were adjoined with polyester #2/0 sutures. Before adjusting the mesh arm tension, the anterior longitudinal ligament was exposed over the promontory and threaded with a polyester #1 suture. Next, 200 ml of physiological saline was injected into the bladder, and the bladder wall was carefully observed using a rigid cystoscope (70º) under various traction pressures of the mesh that were applied by pulling the mesh arm with forceps. When the ideal tension was reached, the mesh arm was attached to the promontory with a thread that had already been passed. During mesh attachment, transvaginal examination was concurrently performed to confirm apex elevation. Even if there was insufficient anterior vaginal wall elevation, the point Aa above −1 cm from the hymen was acceptable. After ligation at the promontory, we confirmed that the mesh arm was tension free laparoscopically and that there was no appearance of a CR cystoscopically. Finally, the mesh was trimmed and buried by closing the peritoneum with polyglactic #2/0 continuous running sutures.

## Results

Table 1 shows the clinical characteristics of the patients at baseline. Fifteen patients with stage 3 POP and five with stage 4 POP were included. Two patients had a history of previous POP repair.

**Table 1:** Baseline patient characteristics

Characteristics	*n* = 20
Age (years)^a^	63 (43–78)
Race^b^ Japanese	20 (100)
Parity^a^	2.0 (1–3)
BMI (kg/m^2^)^a^	23.2 (18.4–29.4)
Menopausal^b^	17 (85)
Hormone replacement therapy usage^b^	0 (0)
Prior hysterectomy^b^	0 (0)
Prior POP surgery^b^ TVM Unknown	2 (10) 1 (5) 1 (5)
Prior continence surgery^b^	0 (0)
Hypertension^b^	6 (30)
Diabetes^b^	2 (10)
Chronic cough^b^	0 (0)
Smoking^b^	0 (0)
Baseline POP-Q stage^b^	
III IV	15 (75) 5 (25)

*BMI* body mass index, *POP-Q* Pelvic Organ Prolapse Quantification, *TVM* tension-free vaginal mesh

^a^Data are expressed as median (range)

^b^Data are expressed as *n* (%) for categorical variables

Table 2 shows the surgical outcomes. All 20 patients underwent concomitant subtotal hysterectomy and one underwent additional cervical amputation because of an elongated uterine cervix. No concomitant anti-incontinence surgery was performed. There were no surgical complications during and after surgery in any patient.

**Table 2:** Operative characteristics

Characteristics	*n* = 20
Time (min)^a^	281 (212–345)
Blood loss (ml)^a^	29 (2–200)
Concomitant procedures at LSC^b^ Subtotal hysterectomy Cervix amputation Oophorectomy Salpingectomy	20 (100) 1 (5) 8 (40) 20 (100)
Complication^b^	0 (0)
Prolapse recurrence (anatomic)^b, c^	4 (20)
Prolapse recurrence (symptomatic)^b, d^	1 (5)
Retreatment^b^	0 (0)

*LSC* laparoscopic sacrocolpopexy

^a^Data are expressed as median (range)

^b^Data are expressed as *n* (%) for categorical variables

^c^POP-Q stage ≥ 2 prolapse

^d^Symptom of bulge according to the Prolapse Quality of Life Questionnaire

**Fig. 1:**
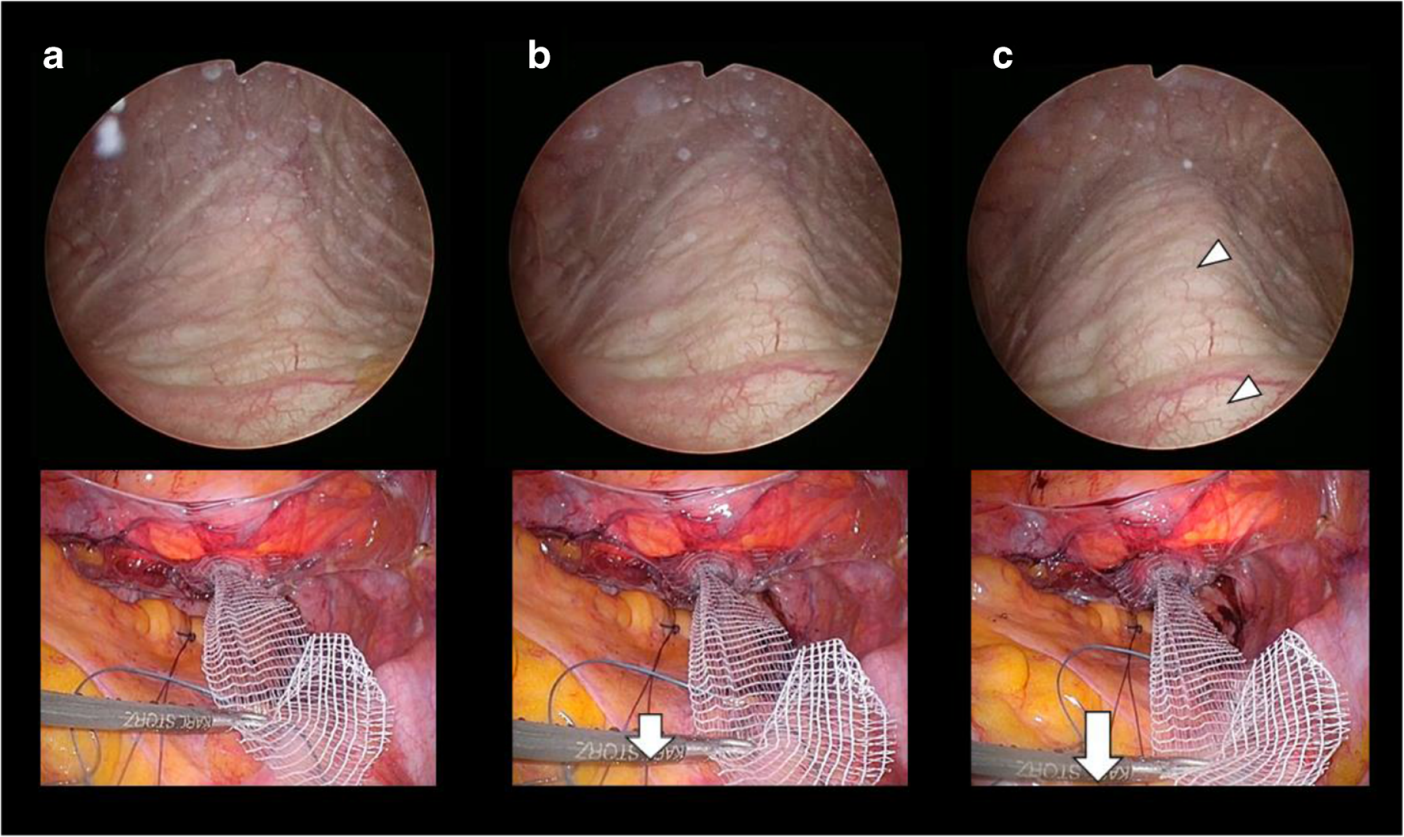
Cystoscopic and laparoscopic findings. **a** No change. **b** Slight elevation in the center of the trigone. **c** A cord-like elevation in the center of the trigone and posterior wall “central road.” The arrowheads indicate the “central road.” The arrows show the direction of tension or force applied on the mesh arms

Cystoscopic findings along with laparoscopic images are shown in Fig. 1. Cystoscopic findings observed while applying the various mesh traction pressures fell into three categories. First, no change was observed on the bladder wall when the vaginal apex was elevated without mesh tension (Fig. 1a). Second, a slight elevation in the center of the trigone was observed when a small amount of traction was applied on the mesh arm (Fig. 1b). Third, the CR finding and bladder neck opening were observed when the mesh arm was tugged powerfully (Fig. 1c). The mesh tension was gradually relaxed after the CR finding was noted, and the CR disappeared accordingly. Subsequently, the mesh arm was attached to the promontory at the point where the CR finding disappeared.

The CR finding and bladder neck opening were observed in all patients when excessive traction was applied on the mesh arm. An anatomically appropriate vaginal apex elevation was achieved precisely when the findings disappeared.

Even after decreasing the pneumoperitoneum pressure, no significant change was observed in the cystoscopic finding, although the mesh tension had slightly diminished.

**Fig. 2:**
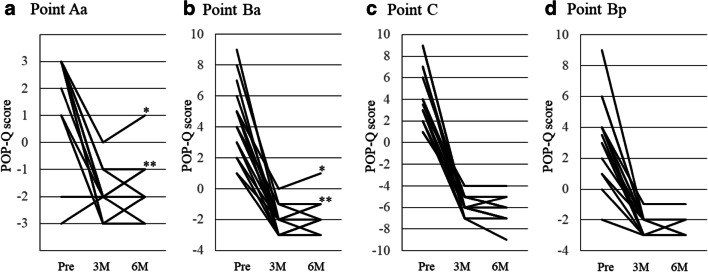
Pre- and postoperative POP-Q scores. The lines represent the pre- and postoperative pelvic organ prolapse quantification (POP-Q) score at points Aa (**a**), point Ba (**b**), point C (**c**) and point Bp (**d**). Pre: before surgery; 3 M: 3 months after surgery; 6 M: 6 months after surgery. *The POP-Q score was +1 in one patient at 6 months after surgery. **The POP-Q score was −1 in three patients at 6 months after surgery

POP-Q was measured at 3 and 6 months after surgery. Although four patients (20%) were diagnosed with anterior wall recurrences, retreatment was not required because the degree of anterior prolapse was POP-Q stage 2 (point Aa; +1 in one and − 1 in three patients) with few symptoms and the apex was sufficiently elevated (point C ≥ –5) in all four patients. In the other 16 patients, all compartments were well repaired (Fig. 2).

**Fig. 3:**
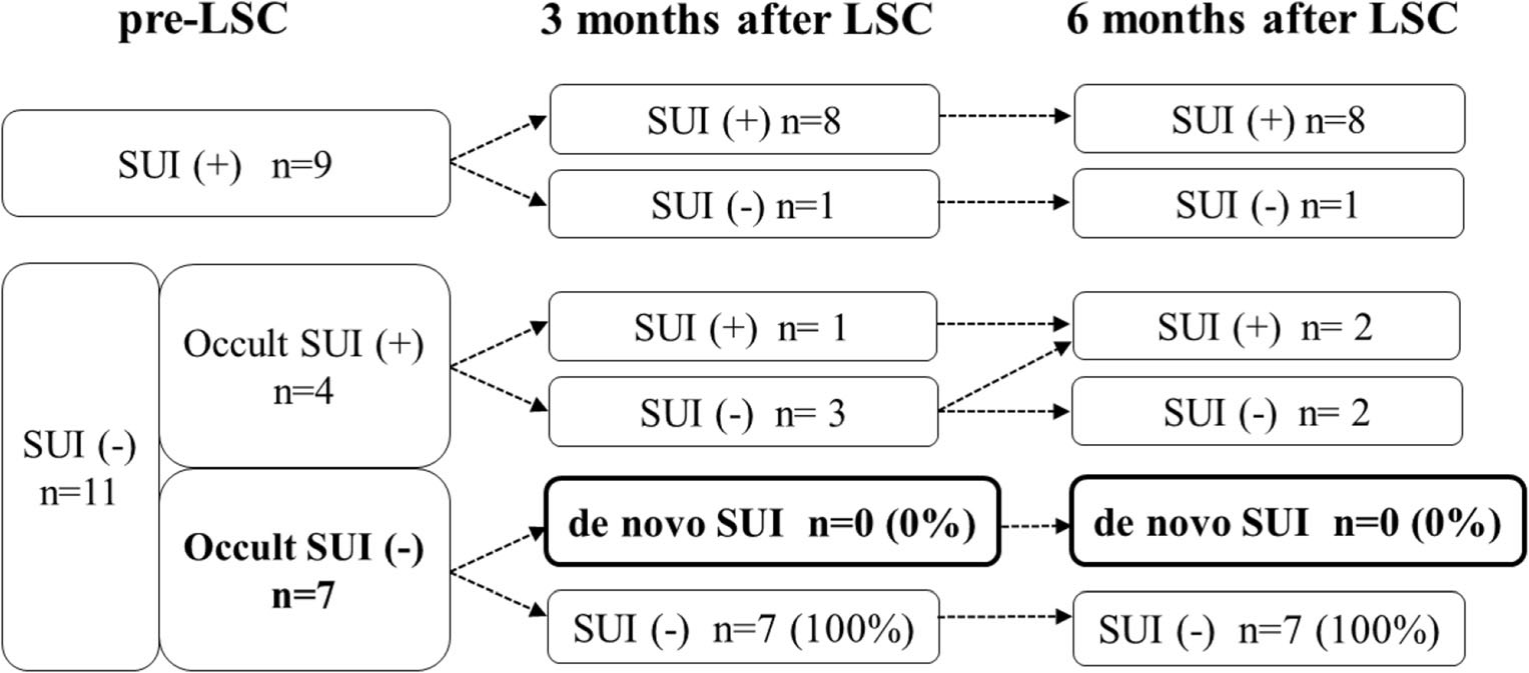
Pre- and postoperative SUI. SUI was assessed based on the International Consultation on Incontinence Questionnaire-Urinary Incontinence Short Form (ICIQ-UI SF). Occult SUI was defined as the presence of SUI after reduction of POP that is confirmed by an interview or a cough test

Among 20 patients, 13 had preoperative SUI (9 and 4 had overt and occult SUI, respectively). Seven patients had no preoperative SUI symptoms and a negative stress test. Postoperative de novo SUI did not occur in those seven patients (Fig. 3).

## Conclusion

To our knowledge, this is the first report exploring mesh tension adjustment using cystoscopy during LSC. This simple method allows the visualization of the bladder wall during mesh adjustment and avoids bladder neck opening due to excessive mesh tension. The limitations of this report were the small number of cases and the short follow-up period. Further investigation with more cases and long-term follow-up is necessary to judge whether this method promotes effective POP repair and de novo SUI reduction.

## Supplementary Information

ESM 1 (MP4 98,895 kb)
